# Mounier-Kuhn syndrome

**DOI:** 10.11604/pamj.2019.33.157.19320

**Published:** 2019-07-02

**Authors:** Badreeddine Alami, Mustapha Maaroufi

**Affiliations:** 1 Department of Radiology and Clinical Imaging, University Hospital of Fez, Fez, Morocco; 2 Laboratory of Clinical Neuroscience, Faculty of Medicine, Fez, Morocco; 3 Department of Biophysics and Clinical MRI Methods, Faculty of Medicine, Fez, Morocco

**Keywords:** Trachea, dilatation, CT-scan

## Image in medicine

A 39-year-old man presented with a productive cough, a history of chronic dyspnea and recurrent lower respiratory tract infections. Physical examination and laboratory tests were unremarkable. A chest X-ray (A) showed an enlarged tracheal diameter (white arrows), with multiple cysts in the lower lobes (red arrows). A computed tomography scan of the chest (B) showed the dilatation of both the trachea and the two main bronchi (white arrows) with multiple diverticulae (blue arrows). It also revealed bilateral cystic bronchiectasis involving both lower lung zones (red arrows). A possibility of Mounier-Kuhn syndrome (MKS) was considered. MKS is a rare condition characterized by recurrent lower respiratory tract infections and tracheobronchial dilation that is due to atrophy of the muscular and elastic tissues in the trachea and main bronchial wall. The patient underwent bronchoscopy which demonstrated tracheal dilation with diverticulae and enlargement of both main bronchi confirming the diagnosis of MKS.

**Figure 1:**
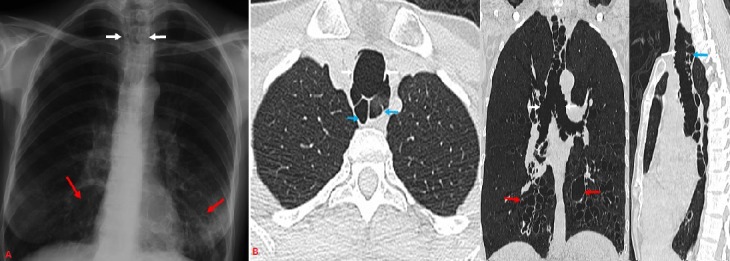
A) a chest X-ray showing an enlarged tracheal diameter (white arrows), with multiple cysts in the lower lobes (red arrows); B) computed tomography scan of the chest showing a dilatation of both the trachea and the two main bronchi (white arrows) with multiple diverticulae (blue arrows). It also revealed bilateral cystic bronchiectasis involving both lower lung zones (red arrows)

